# Rapid Temporal Control of Foxp3 Protein Degradation by Sirtuin-1

**DOI:** 10.1371/journal.pone.0019047

**Published:** 2011-04-20

**Authors:** Jorg van Loosdregt, Diede Brunen, Veerle Fleskens, Cornelieke E. G. M. Pals, Eric W. F. Lam, Paul J. Coffer

**Affiliations:** 1 Department of Immunology, University Medical Center Utrecht, Utrecht, The Netherlands; 2 Center for Molecular & Cellular Intervention, Wilhelmina Children's Hospital, University Medical Center Utrecht, Utrecht, The Netherlands; 3 Department of Cell Biology, University Medical Center Utrecht, Utrecht, The Netherlands; 4 CR-UK Labs and Department of Surgery and Cancer, Imperial College London, Hammersmith Hospital, London, United Kingdom; University of California Los Angeles, United States of America

## Abstract

Maintenance of Foxp3 protein expression in regulatory T cells (Treg) is crucial for a balanced immune response. We have previously demonstrated that Foxp3 protein stability can be regulated through acetylation, however the specific mechanisms underlying this observation remain unclear. Here we demonstrate that SIRT1 a member of the lysine deacetylase Sirtuin (SIRT) family, but not the related SIRTs 2–7, co-localize with Foxp3 in the nucleus. Ectopic expression of SIRT1, but not SIRTs 2–7 results in decreased Foxp3 acetylation, while conversely inhibition of endogenous SIRT activity increased Foxp3 acetylation. We show that SIRT1 inhibition decreases Foxp3 poly-ubiquitination, thereby increasing Foxp3 protein levels. Co-transfection of SIRT1 with Foxp3 results in increased Foxp3 proteasomal degradation, while SIRT inhibition increases FOXP3 transcriptional activity in human Treg. Taken together, these data support a central role for SIRT1 in the regulation of Foxp3 protein levels and thereby in regulation of Treg suppressive capacity. Pharmacological modulation of SIRT1 activity in Treg may therefore provide a novel therapeutic strategy for controlling immune responses.

## Introduction

Protein acetylation is a tightly controlled process reciprocally regulated by lysine acetyl transferases (KATs; also known as histone acetyl transferases) and lysine deacetylases (KDACs; also known as histone deacetylases). The possibility of manipulating immune responses through specific modulation of protein acetylation is being increasingly examined [1]. *In vitro* and *in vivo* studies have demonstrated that treatment with a variety of KDAC inhibitors increases regulatory T cell (Treg) numbers, Treg mediated suppression and decreases disease scores in murine models or arthritis, allograft rejection and colitis [2]–[5]. Recently, several studies have reported Foxp3 acetylation, a transcription factor that is crucial for both Treg development and function [5]–[7]. Foxp3 has been reported to associate with the KATs TIP60 and p300 [5]; [6], while TIP60 and p300 both promote Foxp3 acetylation resulting in both increased Foxp3 protein stability and chromatin binding [5]–[7]. The KDACs: HDAC1, HDAC7, HDAC9 and SIRT1 have all been reported to associate with Foxp3. However, although they have all been shown to impair Foxp3 transcriptional activity or decrease protein stability, none of these KDACs have been reported to directly reduce Foxp3 acetylation [5]; [6]; [8]. Very recently it has been shown that Foxp3 protein levels were increased in SIRT1 knock-out Treg resulting in increased suppressive capacity [9]. However, the molecular mechanisms underlying these observations or the specificity between SIRT family members have not yet been evaluated.

In humans, the class III lysine deacetylases also known as sirtuins (SIRTs) consists of seven members (SIRT1–7). This is a highly conserved gene family encoding nicotinamide adenine dinucleotide (NAD)-dependent protein deacetylases. All SIRTs contain a 250 amino acid core domain that shares 25–60% sequence identity [10]. Individual SIRTs are localized to specific subcellular compartments and have been reported to mediate a variety of cellular processes, including cell differentiation, chromatin remodelling, DNA repair, endocrine signalling, and apoptosis [11]; [12]. Currently little is understood concerning the role of SIRTs in immune responses, although it has been reported that SIRT1 deficient mice are more prone to develop autoimmunity and that Treg specific deletion of SIRT1 increases their immunosuppressive capacity [9]; [13]; [14]. The role for specific SIRT family members in regulating Foxp3 acetylation levels still remains poorly understood.

Recently, we have reported that acetylation abrogates Foxp3 proteasomal degradation through a reduction in Foxp3 poly-ubiquitination levels [5]. As both ubiquitination and acetylation are restricted to lysine residues, a competition model for lysine modification could be envisaged. Here, we demonstrate that SIRT1, but not SIRTs 2–7, co-localizes with Foxp3 in the nucleus and negatively regulates Foxp3 protein levels. SIRT1 deacetylates Foxp3, resulting in increased Foxp3 poly-ubiquitination and proteasomal degradation. Finally, we show that SIRT1 inhibition results in increased FOXP3 transcriptional activity in human Treg. Taken together, these data suggest that specific modulation of SIRT1 activity may provide a therapeutic approach to pharmacologically control immune responses.

## Materials and Methods

### Ethics statement

This study was conducted according to the principles expressed in the Declaration of Helsinki. The Study was approved by the Institutional Review Board of the UMC Utrecht. All participants provided written informed consent for the collection of samples and subsequent analysis.

### Antibodies, DNA constructs and reagents

The following antibodies were used:mouse anti-Foxp3 clone PCH101 for FACS analysis (eBioscience, San diego, CA), rabbit anti-acetyl-lysine (Cell signaling, Danvers, MA), mouse anti-Flag M2, and mouse anti-tubulin from Sigma (Zwijndrecht, The Netherlands), Mouse anti-hemaglutinin (HA) clone 12CA5, and rabbit anti-HSP90 from Santa Cruz Biotechnology (Santa Cruz, CA), (Sigma), HRP-conjugated anti-ubiquitin Enzo Life Sciences (Plymouth Meeting, PA, USA). pMT2 HA-Foxp3, pMT2 HA-Foxp3K22xR, pMT2 Flag-Foxp3 and pRSV-NFATC/A are previously described [5]. Flag-SIRT1–7 constructs have previously been described [15] although we had to re-subclone SIRT5. Cyclohexamide, nicotinamide, and MG132 were purchased from Sigma.

### Cell culture and luciferase assays

HEK293 cells were maintained in DMEM (Invitrogen) supplemented with 8% heat-inactivated FCS, penicillin and streptomycin (Invitrogen) at 37°C and 5% CO_2_. Cells were grown to 50% confluence in six wells-plates (Nunc, Roskilde, Denmark) and transfected with a mixture of 1.5 *µ*g DNA and 7.5 *µ*l PEI overnight, the following day cells were washed twice with PBS and cultured for 24 hours in medium. Cell lysates were separated by SDS-PAGE and analyzed by Western blot. For luciferase assay, cells were transfectied with 1 *µ*g IL–2 promoter luciferase reporter from Panomics (Fremont, CA) 0.5 *µ*g of pMT2–Foxp3, pcDNA3–NFATC/A or 0.5 *µ*g pcDNA3 empty vector and 7 *µ*g pMT2 empty vector and 0.05 *µ*g pRLTK renilla, (Promega, Leiden, the Netherlands) to normalize for transfection efficiency. Cells were transfected in a six-well plate, two days after transfection the cells were washed twice with PBS and lysed in 50 *µ*l passive lysis buffer for 15 minutes, insoluble cell debris was spun down and the supernatant fraction was assayed for luciferase activity using Dual-Luciferase Reporter Assay System (Promega, Leiden, The Netherlands).

### Confocal imaging

#### Localization studies

Transfected HEK293 cells seeded on poly-L-lysine-coated coverslips were washed with PBS before fixation using 3% paraformaldehyde (Merck, Nottingham, United Kingdom) for 10 minutes at 22°C. Cells were pre-incubated with PBS containing 10% normal donkey serum (Jackson Immunoresearch) and 0.5% saponin (Sigma-Aldrich) for 15 minutes. Next, cells were incubated for 60 minutes with mouse anti-Flag-FITC (2.5 µg/ml) and rabbit anti-HA-TRITC 4 µg/ml (Santa Cruz Biotechnology Inc.) in PBS containing 10% normal donkey serum and 0.5% saponin. Cells were washed three times with PBST (0.05% Tween), and mounted in Mowiol 4–88 (Sanofi-Aventis, Paris, France) containing DAPI. Cells were analyzed with a 63x objective on a Zeiss LSM 710 fluorescence microscope (Oberkochen, Germany).

#### Proximity ligation assay

PLA detection was performed according to the manufacturer's protocol (Olink Bioscience, Uppsala, Sweden). In short, HEK 293 cells grown on coverslips were fixed using 3% paraformaldehyde for 10 minutes at 22°C. Subsequently, cells were washed three times with PBS and blocked for 30 minutes at 22°C in PBS containing 10% normal donkey serum, 0.5% BSA, and 0.5% saponin. After blocking, cells were incubated for 60 minutes at 22°C with mouse anti-HA and rabbit anti-Flag antibodies in PBS containing 10% normal donkey serum, 0.5% BSA, and 0.5% saponin. Cells were washed three times with PBST (0.05% Tween) and incubated with the secondary mouse PLUS and rabbit MINUS antibodies for 1.5 hours at 37°C in the dark. Cells were washed three times in PBST before detection of the probe using the *in situ* PLA detection kit (Abnova, Walnut, USA). Cells were analyzed with a 63x objective on a Zeiss LSM 710 fluorescence microscope.

### Immunoprecipitation and Western blot analysis

HEK 293 cells were lysed in a NP40 lysis buffer (0.05 M Tris-HCl pH 7.5, 0.5% Nonidet P40, 0.15 M NaCl, 0.01 M EDTA), immunoprecipitation was performed utilizing anti-flag coupled beads (Sigma). Beads were washed 3x in lysis buffer, boiled and samples were separated by SDS-PAGE, electrophoretically transferred to polyvinylidene difluoride membrane (Millipore, Bedford, MA), and hybridized with antibodies as indicated. Immunocomplexes were detected using enhanced chemiluminescence (Amersham, Buckinghamshire, United Kingdom).

### Generation of iTreg

CD4+CD25- T cells were isolated from human cord blood by magnetic-activated cell sorting (MACS) and cultured in RPMI 1640 supplemented 10% FCS, 100 units/ml penicillin, 100 µg/ml streptomycin, and 5×10^–5^ M 2-mercaptoethanol. Foxp3 expression was induced by culturing the cells for four days in combination with anti-CD3 anti-CD28 beads dynabeads, 300 IU IL-2 and 10 nM TGF-β.

### Quantitative PCR

mRNA was isolated using the trizol according to the manufacturer's protocol (Invitrogen), cDNA synthesis was performed using IScript cDNA synthesis kit (Bio-Rad, Hercules, CA). cDNA samples were amplified using SYBR green supermix (BIO-Rad), in a MyiQ single-color real time PCR detection system (Bio-Rad) according to the manufacturer's protocol. To quantify the data, the comparative Ct method was used [16]. Relative quantity was defined as 2^-ΔΔCt^ and β2-microglobulin was used as reference gene. Primers are listed in Table 1.

**Table 1:** Q-PCR primers.

Gene	Forward primer	Reverse Primer
IL-2	5'-TCCTGTCTTGCATTGCACTAAG-3′	5′-CATCCTGGTGAGTTTGGGATTC-3′
ITK	5′-GTGTTTGCTCCAGATCGTGAG-3′	5′-GGTTGGATCATATTGGGCACAG-3′
cMyc	5′-TCCGGAAGGACTATCCTGCTG-3′	5′-GTGTGTTCGCCTCTTGACATT-3′
ZAP70	5′-TGCCCTTCTTCTACGGCAG-3′	5′-ACGAGCGACAGCACATAGC-3′
JAK2	5′-CAACAGAGCCTATCGGCATGG-3′	5′-GGGGTTTGATCGTTTTCTTTGG-3′
SIRT1	5′-TGCGGGAATCCAAAGGATAA-3′	5′-CAGGCAAGATGCTGTTGCA-3′
Foxp3	5′-TCAAGCACTGCCAGGCG-3′	5′-CAGGAGCCCTTGTCGGAT-3′
β2M	5'-CCAGCAGAGAATGGAAAGTC-3'	5'-GATGCTGCTTACATGTCTCG-3'

### Statistical analysis

Statistical analysis was performed using the Mann-Whitney test (Prism GraphPad Software, San Diego, CA). p<0.05 was considered statistically significant.

## Results

### SIRT1 co-localizes with Foxp3 reducing Foxp3 protein expression levels

Recently, we reported that proteasome-mediated degradation of Foxp3 is inhibited by p300-mediated acetylation [5]. Increased acetylation prevented poly-ubiquitination and subsequently proteasomal degradation in a competition-based manner, as has been described for other transcription factors including RUNX3, SMAD7, and P53 [17]–[19]. To further validate these observations we wished to determine whether there was specificity among sirtuin (SIRT) lysine deacetylase family members in their capacity to regulate both Foxp3 acteylation and ubiquitination.

**Figure 1:**
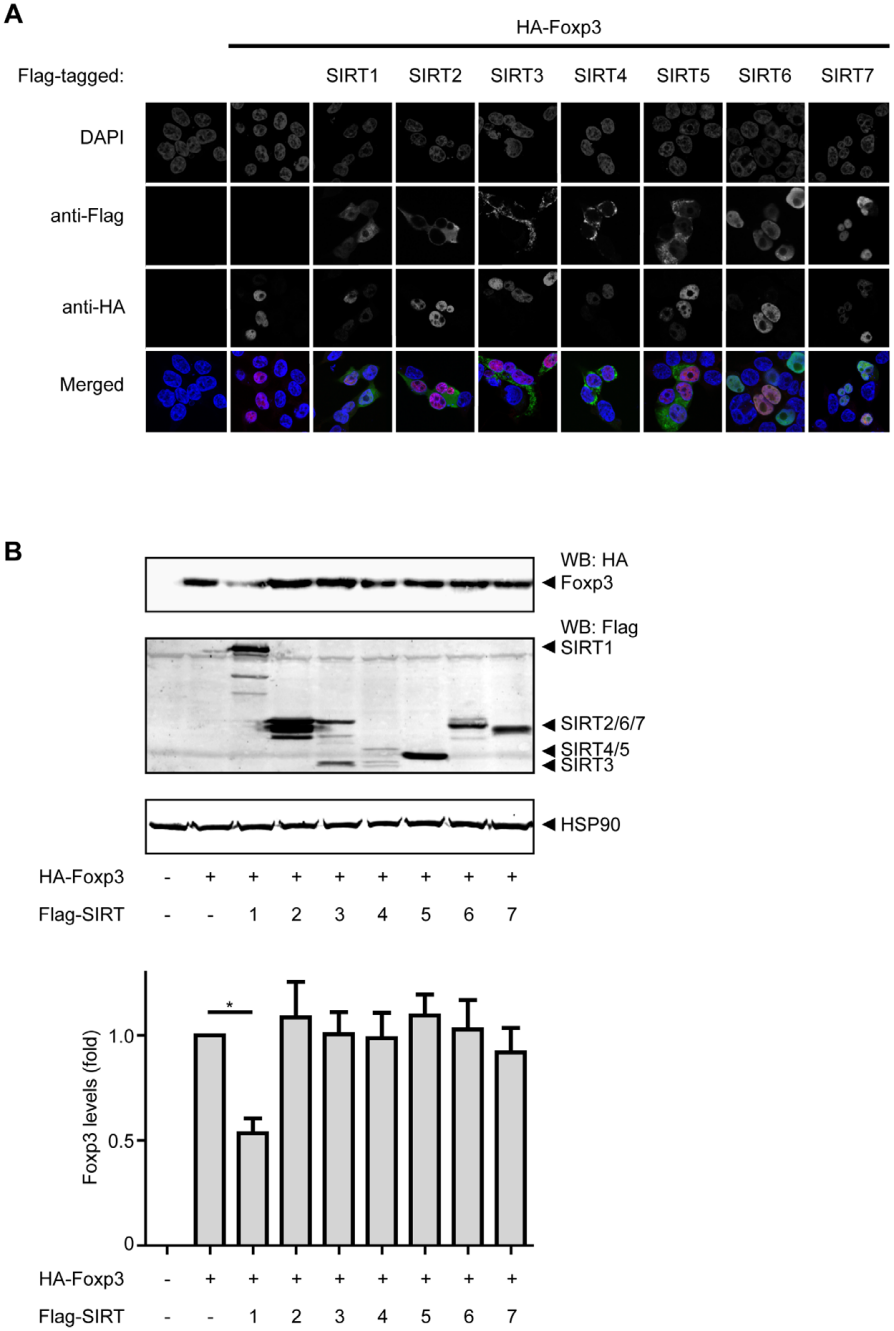
SIRT1 co-localizes with Foxp3 and reduces its expression. (**A**) HEK293 cells that were transfected with HA-Foxp3 and Flag-tagged SIRT1-7. Cells were fixed, permiabilized and tagged proteins were visualized using anti-HA (red) or anti-Flag (green) antibodies. DAPI was used to visualize the nuclei (blue). Figure shows examples of three independent experiments. (**B**) HEK293 cells were transfected with HA-Foxp3 and Flag-SIRT1-7. After 48 hours cells were lysed, and Foxp3 levels were analyzed by Western blotting using anti-HA antibodies. Foxp3 protein levels were quantified and normalized for HSP90 expression. Data show a mean of five independent experiments+ SEM. * P<0.05.

Firstly, to analyze potential interactions between SIRT family members and Foxp3, the subcellular localization of both Foxp3 and SIRTs 1–7 was evaluated. HEK293 cells ectopically expressing HA-tagged Foxp3 and Flag-tagged SIRTs 1–7 were fixed and permeabilized. Subsequently, the localization of Foxp3 and SIRTs 1–7 were analyzed utilizing anti-HA and anti-Flag antibodies. Foxp3 was found specifically localized in the nucleus and this was unaffected by SIRT co-transfection (Figure 1A). Although all SIRTs were expressed to similar levels, we could only detect nuclear localization of SIRT1, 6 and 7. To investigate specifically which SIRTs could have the capacity to decrease Foxp3 expression levels, cells were transfected with both HA-Foxp3 and Flag-SIRTs 1–7, and Foxp3 protein levels were determined using anti-HA antibodies and blots quantified. Co-transfection of SIRT1 reproducibly decreased Foxp3 levels, while SIRTs 2–7 had no observable effect on Foxp3 expression (Figure 1B). Taken together, these data show that SIRT1 specifically co-localizes with Foxp3 and increased SIRT1 expression is sufficient to reduce Foxp3 protein levels.

### SIRT1 directly associates with Foxp3

**Figure 2:**
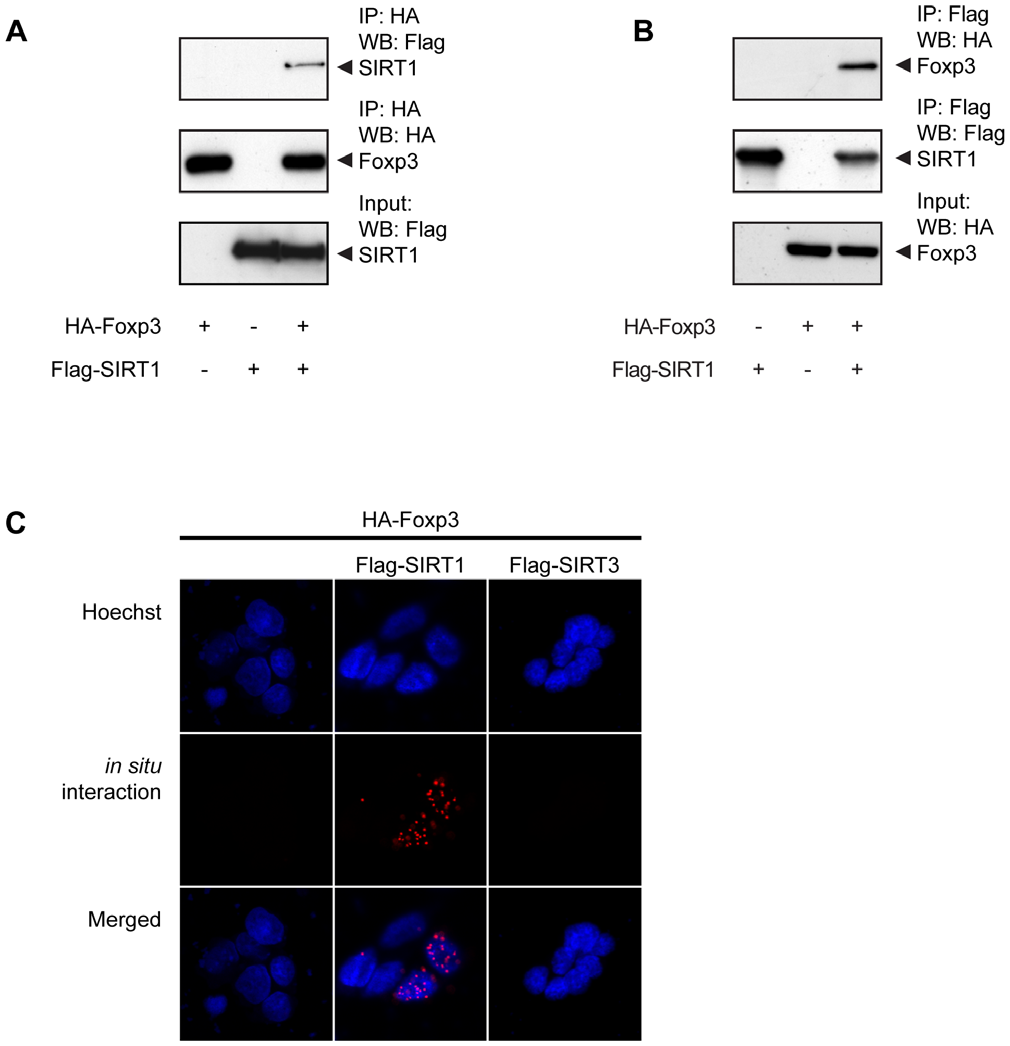
Nuclear association of Foxp3 and SIRT1. (**A**) HEK293 cells were co-transfected with both HA-Foxp3 and Flag-SIRT1, lysed, and Foxp3 was immunoprecipitated and association of proteins was analyzed by Western blotting utilizing anti-Flag antibodies. (**B**) HA-Foxp3 and Flag-SIRT1 transfected cells were lysed, SIRT1 was immunoprecipitated and the association of Foxp3 was assessed as in (**A**). (**C**) SIRT1-Foxp3 interaction in transfected HEK293 cells was visualized using *in situ* proximity ligation assay (PLA). Cells were fixed and protein-protein interactions were visualized utilizing anti-HA and anti-Flag antibodies as described in the Materials and Methods section. Punctate staining (red) indicates Foxp3-SIRT1 interaction as detected by the assay. Nuclei were stained using Hoechst. Representative images from at least three independent experiments are depicted.

To determine whether SIRT1 associates with Foxp3 a co-immunoprecipitation assay was performed. HEK293 cells were transfected with both HA-Foxp3 and Flag-SIRT1, lysates were prepared and Foxp3 or SIRT1 were immunoprecipitated. SIRT1 was detected when Foxp3 was immunoprecipitated and *vice versa*, demonstrating their association (Figure 2A, B). To further verify these results an *in situ* proximity ligation assay (PLA) was performed (see Materials and Methods). Since a PLA signal can only be obtained when the proteins of interest are in extreme close proximity, this technique enables the detection of direct protein-protein interactions in cells. Association of transfected Foxp3 and SIRT1 in HEK293 cells was observed and the interaction was localized specifically in the nucleus (Figure 2C; middle panel). The specificity of this interaction was confirmed since association of Foxp3 with SIRT3 was not observed by PLA (Figure 2C; right panel).

### SIRT1-mediated deacetylation increases Foxp3 poly-ubiquitination

**Figure 3:**
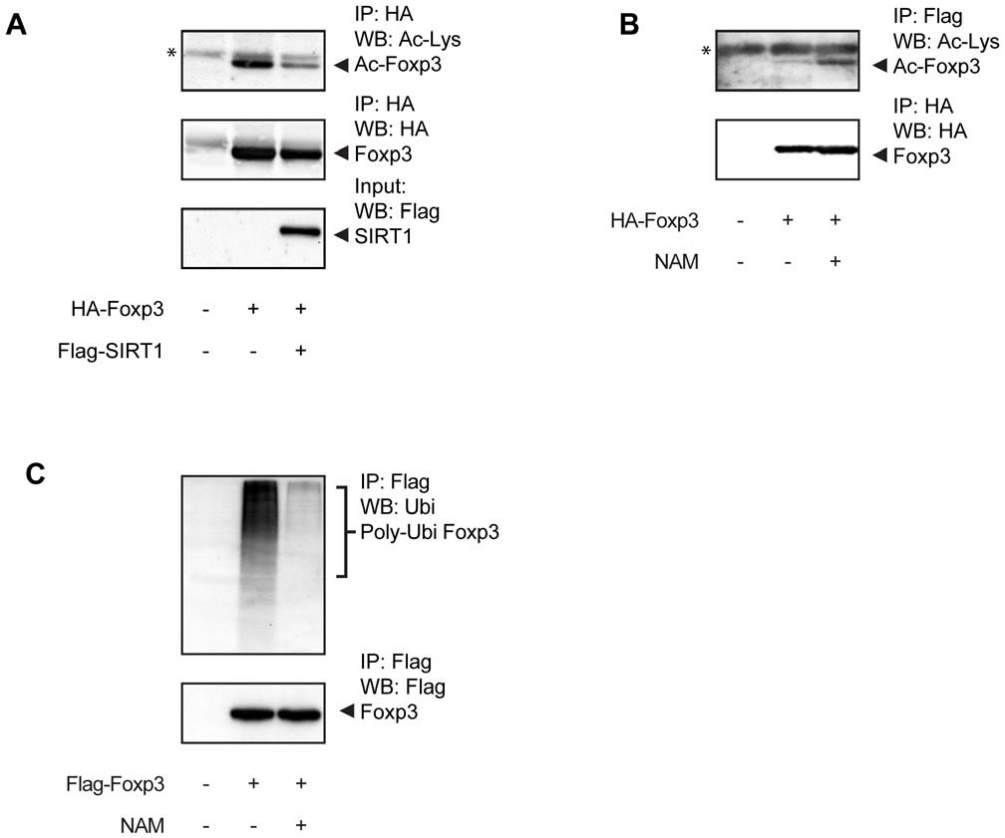
SIRT1-mediated deacetylation increases Foxp3 poly-ubiquitination. (**A**) HEK293 cells ectopically expressing HA-Foxp3 were transfected with Flag-SIRT1. Cell lysates were prepared and HA-Foxp3 was immunoprecipitated. Acetylated Foxp3 was visualized by Western blotting using anti-acetylated lysine antibodies. (**B**) Analysis of Foxp3 acetylation after treatment with 20 mM nicotinamide (NAM) for 16 hours as in (**A**). (**C**) Cells were transfected with Flag-Foxp3 and treated with 20 mM NAM (for three hours), Foxp3 was immunoprecipitated from cell lysates and ubiquitination was analyzed by Western blotting utilizing anti-ubiquitin antibodies. * Indicates aspecific band. Data shown are representative of three independent experiments.

To further evaluate the molecular mechanism underlying the effect of SIRT1 on Foxp3 protein levels, we first determined whether SIRT1 could decrease Foxp3 acetylation levels. HEK293 cells were co-transfected with HA-Foxp3 and Flag-SIRT1, lysates were prepared and HA-Foxp3 was immunoprecipitated, and acetylation was visualized by Western blotting using an anti-acetyl lysine antibody. Levels of Foxp3 acetylation were considerably reduced by SIRT1 co-transfection (Figure 3A). To further validate these data we utilized nicotinamide (NAM), the widely used Sirtuin inhibitor [20]; [21]. NAM treatment increased acetylation of Foxp3 (Figure 3B), demonstrating that SIRTs can indeed modulate Foxp3 deacetylation. Since we previously demonstrated that acetylation of Foxp3 results in increased protein levels by preventing poly-ubiquitination [5], the effect of SIRT inhibition on Foxp3 ubiquitination was also analyzed. Cells ectopically expressing HA-Foxp3 were treated for three hours with the proteasome inhibitor MG132, lysed and Foxp3 was immunoprecipitated. Ubiquitinated Foxp3 was visualized by Western blotting utilizing anti-ubiquitin antibodies. SIRT inhibition dramatically reduced Foxp3 poly-ubiquitination (Figure 3C). Taken together, these data show that SIRT1-mediated deacetylation results in increased Foxp3 poly-ubiquitination.

### SIRT1 regulates Foxp3 proteasomal degradation

**Figure 4:**
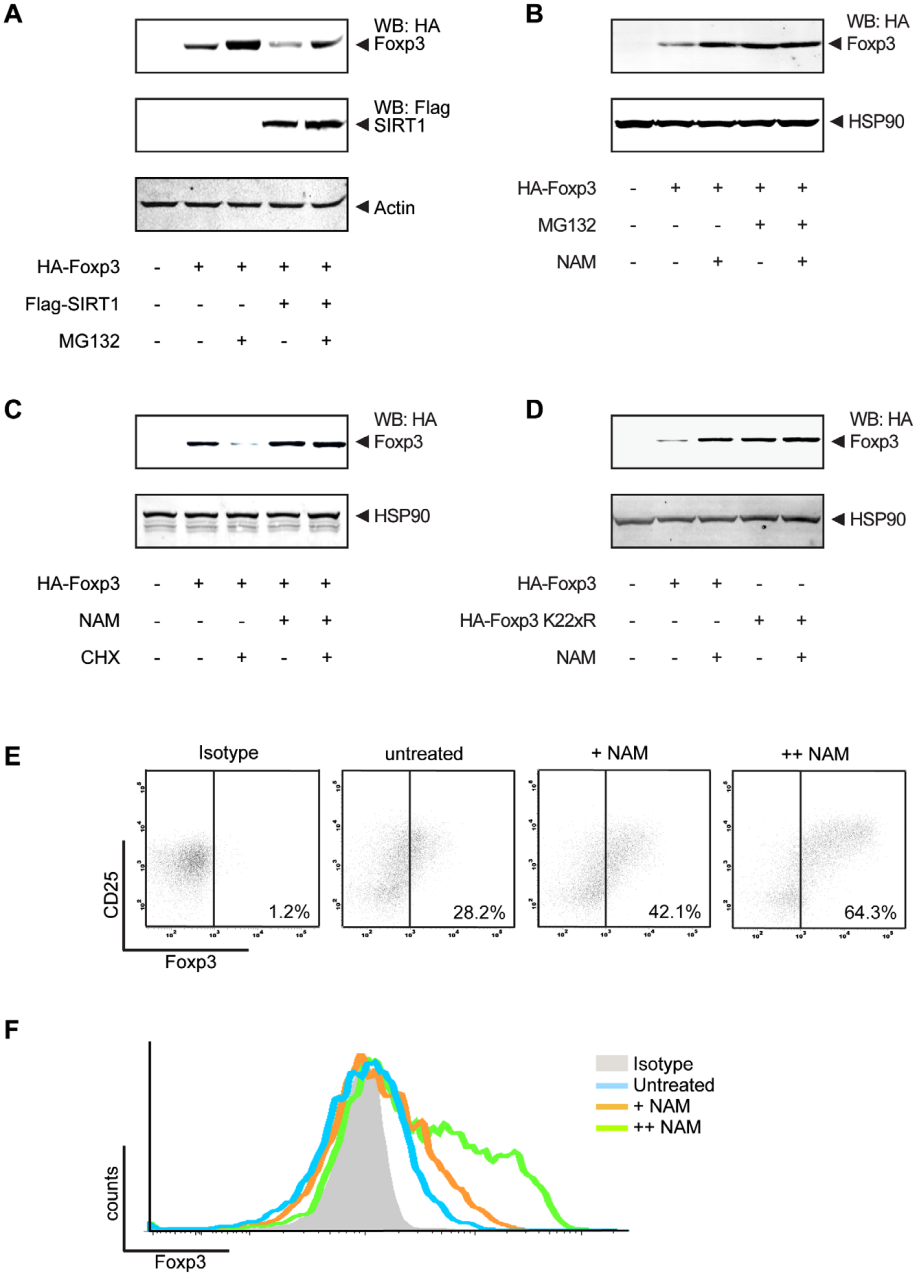
SIRT1 regulates Foxp3 degradation. (**A**) HEK293 cells were co-transfected with HA-Foxp3 and Flag-SIRT1 and treated with 2 µM MG132 for 16 hours. Cells were lysed and Foxp3 protein levels were visualized by Western blotting using anti-HA antibodies. (**B**) Cells were transfected with HA-Foxp3 and treated with 20 mM NAM for 16 hours, Foxp3 protein levels were analyzed by Western blotting using anti-HA antibodies. (**C**) Cells ectopically expressing HA-Foxp3 were treated with both 20 mM NAM and 5 µg/ml cyclohexamide (CHX) for 16 hrs and Foxp3 levels were analyzed by Western botting. (**D**) HA-Foxp3 or a HA-tagged Foxp3 mutant in which all lysines were mutated to argenines (HA-Foxp3 K22xR) was transfected into HEK293 cells. Cells were treated with 20 mM NAM for 16 hours. Cell lysates were prepared and Foxp3 levels were evaluated by immunoblotting for HA and HSP90 as control. (**E**) CD4+ T cells isolated from human PBMC were cultured in the presence of 300 IU/ml IL-2, 2.0 µg/ml anti-CD28, and 1.5 µg/ml plate-bound anti-CD3 and treated NAM (1 or 9 mM). After 4 days, the percentage of Foxp3+CD25+ cells was analyzed by FACS. (**F**) Histogram of the data shown in (**E**). Data are representative of at least three independent experiments.

Since we observed that SIRT1 can decrease Foxp3 deacetylation, thereby increasing its poly-ubiquitination, the effect of SIRT1 on Foxp3 protein stability was further investigated. HEK293 cells were co-transfected with HA-Foxp3 and Flag-SIRT1 and treated with the proteasome inhibitor MG132. After lysis, Foxp3 protein levels were analyzed by Western blotting utilizing anti-HA antibodies. Co-transfection of SIRT1 decreased Foxp3 protein levels, and this was reversed by MG132 treatment (Figure 4A). These data show that the SIRT1-mediated decrease in Foxp3 expression levels is proteasome-dependent**.** To further validate these observations, cells were transfected with HA-Foxp3 and treated with NAM for 16 hours. SIRT inhibition resulted in increased Foxp3 levels as expected (Figure 4B), while MG132 treatment had no additional effect on Foxp3 protein levels. To confirm that SIRT inhibition regulates Foxp3 expression post-translationally, cells were treated with cyclohexamide (CHX), an inhibitor of translation. CHX treatment of cells ectopically expressing HA-Foxp3 resulted in reduced Foxp3 protein levels (Figure 4C). However, there was no effect of CHX-treatment on Foxp3 protein levels when the cells were also treated with NAM, confirming that SIRT1 inhibition increases Foxp3 protein stability.

To confirm that deacetylation of Foxp3 is directly responsible for reduced proteasomal degradation we generated a Foxp3 mutant in which all lysines had been mutated (HA-Foxp3 K22xR), thereby preventing both acetylation and ubiquitination of Foxp3. Cells were transfected with wild-type Foxp3 or Foxp3 K22xR and subsequently treated with NAM. Foxp3 K22xR protein levels were increased compared to wild-type presumably since the Foxp3 K22xR mutant can no longer be poly-ubiquitinated (Figure 4D). Treatment with NAM again resulted in increased protein levels of wild-type Foxp3, however Foxp3 K22xR expression levels remained unaffected by SIRT inhibition.

To determine whether SIRTs can also regulate Foxp3 protein expression in human CD4+ Treg, CD4+ cells from human PBMC were cultured in the presence of IL-2, anti-CD3 and anti-CD28 to generate iTreg as previously described [5]. iTreg were treated with NAM and the percentage of CD4+Foxp3+ T cells, as well as Foxp3 protein levels per cell were determined by FACS. NAM treatment resulted in both increased CD4+Foxp3+ cell numbers as well as increased Foxp3 protein levels per cell (Figure 4E, F). Taken together, these data show that SIRT-mediated deacetylation increases proteasome mediated Foxp3 degradation in Treg.

### SIRT1 activity inhibits Foxp3 transcriptional output

**Figure 5:**
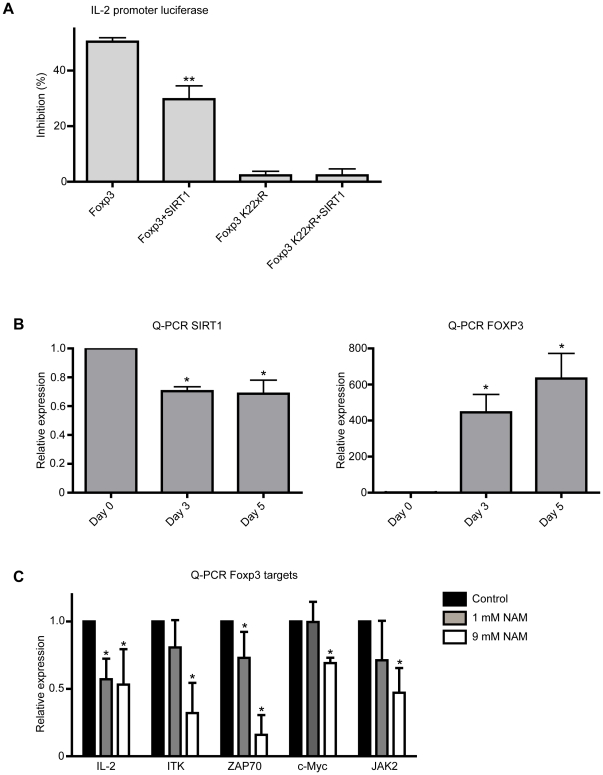
SIRT1 inhibition increases Foxp3 transcriptional output. (**A**) Utilizing an IL-2 promoter reporter, Foxp3 transcriptional function was analyzed. HEK293 cells were transfected with an IL-2 promoter luciferase reporter, NFAT, Foxp3, Foxp3 K22xR and SIRT1, cells were lysed and assayed as described in the Materials and Methods. Luciferase values were normalized for co-transfected renilla to correct for transfection efficiency. (**B**) iTregs were generated by culturing human CD4+CD25- human T cells with anti-CD3 and anti-CD28 coupled beads, in combination with 10 ng/ml TGF-β and 300 U/ml IL-2 for 5 days. At day 0, 3, 5 cells were harvested and mRNA expression of SIRT1 and Foxp3 was analyzed by qRT-PCR. (**C**) Freshly isolated human CD4+ T cells were cultured in the presence of 300 U/ml IL-2, anti-CD3 and anti-CD28 and 0, 1 or 9 mM NAM for three days. mRNA expression of multiple transcriptional targets of Foxp3 was analyzed by qRT-PCR. Data are representative of at least three independent experiments, * P<0.05, ** P<0.01.

To determine whether SIRT1-mediated Foxp3 deacetylation also results in impaired Foxp3 transcriptional output an IL-2 promoter reporter assay was performed. HEK293 cells were transfected with IL-2 promoter luciferase, NFAT, Foxp3 and SIRT1 as previously described [5]. Transfection of Foxp3 resulted in suppression of IL-2 promoter activity (Figure 5A). Co-transfection with SIRT1 significantly impaired Foxp3 mediated repression of the IL-2 promoter. To control for a direct effect of SIRT1 on the IL-2 promoter reporter, luciferase activity was analyzed utilizing the Foxp3 K22xR acetylation-deficient mutant. Foxp3 K22xR was unable to inhibit reporter activity, and this was unaffected by SIRT1 co-transfection. These data further validates the observation that Foxp3 deacetylation results in reduced Foxp3-mediated transcriptional repression. Since we have established that SIRT1 decreased Foxp3 protein levels through proteasomal degradation Foxp3, SIRT1 mRNA expression levels were analyzed during Treg induction. Treg were induced by stimulating CD4+CD25- cells from cord blood peripheral blood mononuclear cells (PBMC) using anti-CD3 anti-CD28 coupled dynabeads in combination with IL-2 and TGF-β for five days. Cells were isolated at day 0, 3, and 5, and SIRT1 and Foxp3 mRNA expression were analyzed by qRT-PCR (Figure 5B). Foxp3 mRNA levels were dramatically upregulated during iTreg differentiation, as expected. Furthermore, a significant decrease of SIRT1 mRNA at both day three and five was observed. We next analyzed whether increased Foxp3 protein levels by SIRT inhibition also results in increased Foxp3 transcriptional activity. Human CD4+ cells were isolated from PBMC and differentiated towards Foxp3+ iTreg in the presence or absence of NAM. mRNA expression of multiple Foxp3 transcriptional targets (IL-2, ITK, Zap70, c-Myc, Jak2) were analyzed [22]. Treatment with NAM reduced mRNA levels of all Foxp3 transcriptional targets in a dose-dependent manner (Figure 5C). Taken together, these data suggest that increased Foxp3 protein levels by SIRT1 inhibition also results in increased suppression of multiple Foxp3 transcriptional targets in iTreg.

## Discussion

Stable expression of the transcription factor Foxp3 is essential for the development and function of Treg [23]–[26]. Although TCR activated T cells can transiently express Foxp3 protein, these cells lack suppressive capacities [27]; [28]. While many studies have focused on the regulation of *Foxp3* promoter activity, proteasome mediated regulation of Foxp3 expression levels has not yet been extensively investigated. Recently, we established that Foxp3 acetylation increases protein levels, and that deacetylation by KDACs decreases Foxp3 protein expression through increased proteasomal degradation [5]. Here we demonstrate that SIRT1, but not SIRTs 2–7, co-localizes with Foxp3 in the nucleus. SIRT1 can specifically regulate Foxp3 deacetylation, resulting in increased Foxp3 poly-ubiquitination and reduced Foxp3 protein levels. In addition, we show that SIRT inhibition results in both increased Foxp3 protein levels and transcriptional output in human iTreg. Taken together, these data provide novel insights into regulatory mechanisms modulating Foxp3 protein expression and thereby Treg numbers and suppressive capacity.

Several classes of KDAC inhibitors are currently being used as anti-inflammatory agents [1]. We and others have recently reported that inhibition of KDAC activity utilizing broad-spectrum KDAC inhibitors, including TSA, SAHA, Tubacin, BML-210, MS-275, SB, and Bufexmac can increase Treg-mediated suppression in *in vitro* suppression assays [4]; [5]; . Treatment with TSA, SAHA, and VPA have also been shown to reduce colitis, and increase cardiac and island allograft survival *in vivo* in mouse models [2]–[4]. Importantly, Beier *et al*. recently reported that in both murine *sirt1* knock-out Treg, and Treg in which SIRT1 was specifically inhibited utilizing SIRT1 inhibitor EX-527**,** Treg mediated suppression was increased, resulting in increased cardiac allograft survival [9]. However, the specificity if SIRT family members for Foxp3 and the molecular mechanisms underlying these observations were not evaluated. Our data support the observations of Beier, *et al.* and provide a molecular mechanism for the increased Treg mediated suppression *in vivo*. In contrast, it was reported that mice with a global deletion of *sirt1* are more prone to develop autoimmunity [13]; [14]. Removing SIRT1 in all cell types may account for the difference in phenotype, since here SIRT1 could potentially also modulate processes of DC mediated antigen presentation, T cell maturation or effector T cell activity.

Here we demonstrate that treatment of human CD4+ T cells with SIRT inhibitor nicotinamide increases the number of CD4+Foxp3+ Treg as well as the amount of Foxp3 per cell by impaired Foxp3 degradation, increasing Treg suppressive capacity. Nicotinamide, also known as vitamin B3, is a water-soluble vitamin and its use in the treatment of auto-immunity would therefore be cheaper and relatively safe compared to other commercially available KDAC inhibitors. In several human trials nicotinamide treatment has already shown to significantly reduce the development of type 1 diabetes [30]; [31]. In addition, nicotinamide treatment of patients with arthritis has been observed to result in disease remission [32]; [33]. IPEX patients have an enhanced susceptibility for diabetes type 1 and Treg adoptive transfer impairs diabetes in NOD mice [34], while depletion of Treg in collagen-induced arthritis mice hastens the onset of disease [35]. With this in mind, it is interesting to speculate that the positive effect of nicotinamide observed in current patient trials could be Treg mediated [36]; [37].

As previously mentioned, activated T cells can also transiently upregulate Foxp3 expression levels [27]. CD4 positive T cells stimulated with phorbol 12-myristate 13-acetate (PMA) and Ca^2+^ ionophore, or anti-CD3 and anti-CD28, transiently express both Foxp3 mRNA and protein [27]; [38]; [39]. In general, these Foxp3 positive cells do not develop a Treg phenotype and are unable to suppress proliferation or cytokine production of co-cultured T cells [27]; [38]. However, a small percentage of stimulated T cells express high and stable levels of Foxp3 and have also been demonstrated to be immune suppressive [27]; [28]. Murine Treg in which Foxp3 is conditionally deleted, lose their suppressive phenotype resulting in the production of Th1 cytokines[26]. These observations underscore the prevailing dogma that stable, high expression of Foxp3 is essential for maintaining the Treg phenotype, and stabilization of Foxp3 expression levels is a critical step for the development of induced Treg. It has recently been shown that SIRT1 is significantly upregulated in activated T effector cells, while in Treg SIRT1 is downregulated upon activation [9]. Since we show that SIRT1 mediated deacetylation of Foxp3 results in impaired protein stability, we propose a model in which Foxp3 acetylation may provide a post-translational “switch” allowing the development of iTreg. In this model, T cell activation results in expression of Foxp3 which will be stable and high when Foxp3 is acetylated, resulting in the development of suppressive iTreg. However, in the presence of active SIRT1, as reported in activated T effector cells [9], T cells will not develop a Treg phenotype since Foxp3 will be deacetylated, subsequently poly-ubiquitinated and degraded by the proteasome. Therefore control of SIRT1 activity may be a defining factor for the initiation of a Foxp3-dependent transcriptional program and the development of Treg.

Taken together, we have demonstrated that SIRT1 but not SIRTs 2–7 directly deacetylates Foxp3, resulting in increased Foxp3 poly-ubiquitination and proteasomal degradation, thereby abrogating Treg functionality. Our findings suggest that manipulation of SIRT1 levels or activity may provide a novel therapeutic strategy to control (inappropriate) immune responses.
